# Management of childhood diarrhea among private providers in Uttar Pradesh, India

**DOI:** 10.7189/jogh.06.010402

**Published:** 2016-06

**Authors:** Christa L Fisher Walker, Sunita Taneja, Laura M. Lamberti, Amnesty Lefevre, Robert Black, Sarmila Mazumder

**Affiliations:** 1 Johns Hopkins Bloomberg School of Public Health, Department of International Health, Baltimore, MD, USA; 2 Society for Applied Studies, New Delhi, India

## Abstract

**Background:**

In Uttar Pradesh (UP), India, a new initiative to introduce zinc and reinvigorate ORS for diarrhea treatment in the public and private sectors was rolled out in selected districts. We conducted an external evaluation of the program that included assessing the knowledge and practices of private sector providers 6 months after the initial program rollout.

**Methods:**

We conducted interviews and direct observations among a randomly selected group of formal and informal private sector providers in 12 districts of UP. We calculated summary statistics for reported provider characteristics, diarrhea treatment knowledge and preferred treatments, as well as the treatments advised during consultation with a child with diarrhea.

**Results:**

We interviewed 232 providers, of whom 67% reported receiving a diarrhea treatment training/drug detailing visit. In the interview, 14% of providers reported prescribing zinc to all children with diarrhea and 36% reported prescribing zinc to more than half of diarrhea cases. During direct observation, ORS and zinc were prescribed by 77.3% and 29.9% of providers, respectively. Treatments other than zinc and ORS were also commonly prescribed, including antibiotics (61.9%) and antidiarrheals (17.5%).

**Conclusion:**

Adequate treatment of childhood diarrhea with zinc and ORS remains a challenge among private sector providers in rural UP, India. Additional training and knowledge transfer activities are needed to curb the overprescription of antibiotics and antidiarrheals and to increase the confidence of private providers in advising zinc and ORS. In addition, policymakers and program implementers must ensure collaborative efforts to target and meaningfully engage informal private providers who play a major role in childhood diarrhea treatment in hard–to–reach areas.

Diarrhea is a leading cause of morbidity and mortality among children less than 5 years of age in low– and middle–income countries [1]. Most diarrheal deaths can be prevented by the simple and effective treatment regimen currently recommended by the World Health Organization, which includes Oral Rehydration Salts (ORS) to prevent and treat dehydration, zinc supplementation for 10–14 days, and continued feeding [2]. The availability of ORS has been widespread in India since the 1980s, and yet it is only used to treat one quarter of diarrheal episodes [3]. Similarly, despite the endorsement of zinc by the Indian Academy of Pediatrics (IAP) in 2004 and 2006 and adoption by the Indian government [4,5], it was not widely available in the public or private sectors in UP prior to implementation of the Diarrhea Alleviation through Zinc and ORS Therapy (DAZT) program in 2011.

In UP, caregivers of children with diarrhea more commonly seek care from private as opposed to public sector providers [6]. The private sector is comprised of providers with formal medical degrees and those practicing in the informal sector, many of whom do not have a license to practice and thus are not recognized by government. Evaluations of private sector providers in India have found that even informal providers are capable of delivering services of relatively high quality for basic medical care if knowledge and competency are high [7]. However, a recent provider assessment using surveys and patient vignettes in Bihar, India found a considerable gap between knowledge and practice with regard to the treatment of pediatric diarrhea [8]. Compared to ORS, zinc is a relatively new addition to the advised childhood diarrhea treatment protocol, and there is a dearth of available evidence on the acceptability of zinc among practitioners in rural areas, many of whom are removed from formal training resources and the influences of pediatric associations promoting national guidelines. In rural India, where up to 80% of children are brought to the private sector for care [9] and informal providers may outnumber qualified physicians 10 to 1, it is difficult to access high quality and consistent care without addressing the importance of the private sector in scaling–up adequate diarrhea treatment [7].

We present the results of a private sector provider assessment conducted in Uttar Pradesh after the rollout of the DAZT program, which aimed to improve diarrhea treatment for children under 5 years of age. The aim of this assessment was to characterize the childhood diarrhea treatment knowledge and practices of both formal and informal private sector providers approximately one year after project roll–out.

## METHODS

### DAZT program description

DAZT was a 4–year project (2011 – 2014) supported by the Bill and Melinda Gates Foundation, which aimed to enhance the uptake of zinc and ORS in rural UP, India. The DAZT project aimed to increase the coverage of ORS and zinc for treatment of diarrhea among children <5 years of age in 12 selected districts. The Johns Hopkins School of Public Health (JHSPH), in collaboration with the Society for Applied Studies, conducted an independent evaluation of the project activities in both the public and private sectors.

The overall goal of the private sector activities, which were led by FHI360, was to increase ORS and zinc prescribing by both formal and informal private sector providers. Formal providers included those who had completed government–recognized medical degrees (MD/MBBS). Informal providers included those with no medical training or certificates in traditional *Ayush* (Ayurveda, Yoga & Naturopathy, Unani, Siddha and Homeopathy) medicine. To target both groups, FHI360 used a two prong “push – pull” strategy. The “push” focused on changing diarrhea prescription practices among key opinion leaders through routine drug detailing for informal sector providers and formal training sessions for practicing physicians. To execute the “pull” component of the strategy, FHI360 recruited and trained staff from non–governmental organizations (NGOs) and private pharmaceutical companies in adequate childhood diarrhea treatment with ORS and zinc. The trained staff visited the practices of rural informal private sector providers to promote and sell ORS and zinc. By providing face–to–face meetings that included a short information video and information materials, FHI360 created demand for appropriate diarrhea treatment.

### DAZT evaluation

The external evaluation of the DAZT program aimed to assess the coverage, quality and cost–effectiveness of implementation efforts through population–based household surveys and provider assessments. Additional details of the program and the results of the household coverage surveys have been published previously [10]. The provider assessment was timed to provide critical information on provider knowledge and behavior early enough to inform and enable programmatic adjustments.

### Sample size

We generated a probability proportional to size (PPS) random sample of 29 tehsils ( ~ 50% of all tehsils) across the 12 districts in UP. By PPS sampling, the proportion of tehsils sampled from each district was equal to the proportion of the private provider population operating in that district relative to the total informal provider population across the 12 districts. Using a sampling frame of providers identified by FHI360 during implementation of the “push – pull” strategy, we randomly selected 8 private providers per tehsil to achieve the required sample size of 232. The sample size requirement was calculated assuming zinc prescribing of 20% at the time of the survey and accounting for 10% margin of error, a design effect of 1.365 (personal communication, S. Taneja) and 15% refusal. Formal and informal providers were sampled as one unit.

### Data collection

We conducted the provider assessment from June –July, 2012, one year after roll–out of the program in June 2011. The survey instruments were developed based on previous surveys conducted by the investigators in similar populations. The survey was carried out by Mindfield Research, an experienced research firm based in New Delhi. The team was comprised of interviewers with previous interview experience who were fluent in both English and Hindi and comfortable interviewing providers. We conducted training of the survey team in New Delhi over the course of 3 days. Training included a thorough review of the survey protocol and survey tools, as well as pilot testing of the interview and observation forms using mock interviews and mock observations.

The trained survey teams visited the selected tehsils (administrative regions denoting sub–districts) according to a preset schedule. Interviewers identified the location of selected providers’ shops/clinics and visited multiple times on the given day in an effort to find the provider. In the case where a provider was not located, the interviewer asked at least 3 other providers and/or community leaders about the whereabouts of the specified provider. If the interviewer was unable to locate the provider by the end of the day, the identified provider was dropped and replaced by the next randomly selected provider on the list who was from the same tehsil but not the same village; this methodology was employed to avoid biasing the sample towards easily accessible providers at the village–level.

Interviewers informed selected providers that the purpose of the visit was to observe the provider treating a child with diarrhea and to subsequently interview the provider about the diarrhea treatment practices typically provided. Interviewers obtained informed written consent and then waited for a caregiver of a child 2–59 months of age to seek care for diarrhea. Standard practice was to conduct the observation before the interview so questions asked during the interview would not bias the treatment provided during the observation. Prior to the observation, the interviewer also obtained verbal consent from the caregiver of the sick child. The interviewer remained a silent observer during the provider’s interaction with the caregiver and child and used a standardized form to log questions on the history of the episode and treatments recommended and/or treatment referrals. The observation lasted approximately 30 minutes.

The interview portion of the assessment lasted approximately one hour and included short vignettes describing children with a range of diarrheal episode symptoms indicative of varying degrees of severity and comorbidities. For each vignette, the provider was asked whether he would refer or treat the child and, if he would treat, he was asked to describe the advised treatment regimen in detail. The survey form was designed to collect categorical responses, including the option of “other,” in which case the interview recorded the exact detailed response given by the provider. The interview also consisted of questions on diarrhea treatment knowledge and practices and access to routinely available ORS and zinc supplies. To ensure confidentiality, all interviews and observed treatment exercises took place in private locations in the presence of survey team member(s) and the provider alone.

After each day of fieldwork, the survey forms were double checked by the supervisor and incomplete entries or logical errors were corrected by contacting the provider immediately. This process ensured that all final forms were complete and free of logical errors prior to photocopying and data entry. The completed surveys were photocopied, with one copy sent via a secure courier to the data entry team at the Society for Applied Studies in New Delhi and one copy remaining with the survey team in the field. If inconsistencies were identified during the data entry process, the survey team attempted to clarify the issue by revisiting the provider if possible.

We received ethical approval from the Johns Hopkins University Bloomberg School of Public Health Institutional Review Board (IRB) and the Society for Applied Studies Ethics Review Committee. All providers signed a full informed consent document. All caregivers of observed children gave verbal consent.

### Statistical methods

We included private sector providers from both the formal and informal sectors, representing the breadth of the private sector DAZT program in UP. The survey was not designed to detect differences between informal and formal sector providers. However, we conducted *t* tests and χ^2^ tests to determine if there were differences in the sociodemographic characteristics of the two groups. We also compared exposure to drug seller informational visits about diarrhea and recall of having seen a video on diarrhea treatment and/or posters promoting zinc and ORS in the last 6 months.

Although interviews were conducted for all participating providers, observations were not available for those who were not visited by a child with diarrhea on the day of the assessment. To assess the generalizability of providers who completed both observations and interviews to those who only completed interviews, we conducted *t* tests and χ^2^ tests of basic sociodemographic characteristics and program exposure and found no differences (data not shown). We therefore combined both sets of providers for all subsequent analyses on interview data. We generated summary statistics for providers’ responses to the clinical vignettes during the interview and adherence to the current WHO and IAP guidelines for childhood diarrhea treatment during the observed consultation [2,5]. All statistical analyses were conducted using Stata 12.0 software [11].

## RESULTS

**Figure 1:**
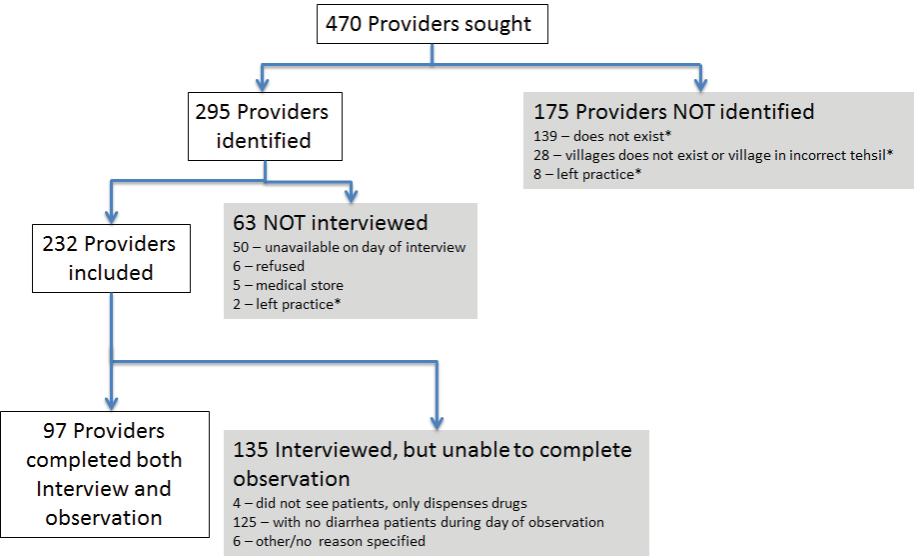
Study sample. Asterisk indicates that the information collected from village administrative heads, other providers, and local shops.

Of the 295 private sector providers included in the sample, 232 (78.6%) agreed to participate in the assessment. Of these, 97 were included in both the interview and the observation and 135 provided information in the interview alone (**Figure 1**). In **Table 1**, we present an overview of the demographics and training of the providers included in our assessment stratified by the informal and formal sector. We also present an overview of the demographics and training of the providers included in our assessment stratified by informal vs formal sector. Among all interviewed providers, 30.6% had a formal medical degree (including MBBS/MD, pharmacy, and nursing) and 69.4% were practicing as part of the informal sector with either Ayurvedic/homeopathic training (21.1%) or no recognized degree or training program (48.3%).

**Table 1:** Comparison of the characteristics of informal and formal providers

Characteristics	Informal providers, n = 161 (%)	Formal providers, n = 71 (%)	P–value
Proportion male	161 (100.0)	71 (100.0)	–
Mean age in years (SD)	43.6 (12.2)	38.9 (11.4)	0.006
Mean years of education (range)	13.1 (3.0)	15.6 (1.4)	<0.001
Proportion who sell drugs/ medicines	150 (93.2)	71 (100.0)	0.024
Proportion who consult and provide a diagnosis for patients	146 (90.7)	60 (84.5)	0.169
Mean years working as a private provider	14.5 (10.6)	11.7 (10.4)	0.059
Mean number of days worked in the last week (SD)	6.4 (1.4)	6.5 (1.1)	0.597
Proportion who recalled an informational visit in past 6 months by any source who spoke about pediatric diarrhea treatment	111 (68.9)	44 (62.0)	0.299
Proportion who recalled seeing a video about diarrhea treatment in last 6 months	56 (34.8)	21 (29.6)	0.438
Proportion who have seen posters advertising the use of zinc for the treatment of diarrhea in last 6 months	136 (84.5)	58 (81.7)	0.598

SD – standard deviation

Ninety–five percent of providers reported prescribing drugs/medications but only 89% reported providing diagnosis or patient consultation; the remaining 11% only sold drugs/medications. In the previous 6 months, 155 providers (67%) reported they had been visited by someone who provided training/information about diarrhea treatment. In addition, 84% of private providers reported seeing posters advertising zinc for diarrhea treatment. There were no differences between formal and informal sector providers with regard to having been visited by a drug seller or having seen a video about diarrhea treatment in the last 6 months, nor were there any difference between the two groups in the proportion who had seen a billboard advertising zinc and ORS. Because the survey was not designed to compare provider types and given there were no differences in these programmatic indicators, we did not stratify by formal/informal in subsequent analyses.

In **Table 2**, we summarize provider responses on the diarrhea treatments typically recommended during five case vignettes. For simple acute diarrhea (5 loose, watery stools/d for 3 days), providers reported recommending ORS (68.1%) and antibiotics (65.9%) most often. However, the proportion reportedly prescribing ORS decreased for the remaining four scenarios, each of which represented more severe diarrhea. For simple acute diarrhea, 35.8% of providers reported prescribing zinc. In all five scenarios, providers reported advising antibiotics more frequently than zinc. As the severity of the case vignette increased with either signs of dehydration, duration of illness, or accompanying signs and symptoms, providers more frequently reported referral in lieu of any type of treatment, including life–saving ORS.

**Table 2:** Treatment practices reported by private providers in interview (n = 232)

Description of a child brought to you with:	Treatments typically prescribed (No, %)*							Only refer (not treat)
**ORS**	**Sugar – salt solution**	**Increased fluids**	**Zinc**	**Antibiotics**	**Antidiarrheals**	**IV**
5 loose/watery stools per day for 3 days with no signs of dehydration	158 (68.1)	14 (6.0)	16 (6.9)	83 (35.8)	153 (65.9)	50 (21.6)	1 (0.4)	43 (18.5)
5 loose/watery stools per day for 3 days with sunken eyes and lethargic	107 (46.1)	7 (3.0)	9 (3.9)	64 (27.6)	103 (44.4)	24 (10.3)	54 (23.3)	106 (45.7)
4 loose/watery stools per day for 15 days	97 (41.8)	9 (3.9)	7 (3.0)	61 (26.3)	82 (35.3)	25 (10.8)	17 (7.3)	122 (52.6)
The presence of blood in the stools	98 (42.2)	5 (2.2)	10 (4.3)	59 (25.4)	130 (56.0)	40 (17.2)	11 (4.7)	91 (39.2)
Fever, fast breathing, and 3 loose, watery stools per day for 3 days	79 (34.1)	5 (2.2)	11 (4.7)	49 (21.1)	93 (40.1)	21 (9.1)	5 (2.2)	128 (55.2)

ORS – Oral rehydration salts

*Multiple responses accepted.

When providers were asked specifically about willingness and frequency of prescribing zinc and ORS, a small percentage reported prescribing zinc to all patients (14%), and an additional 36% reported prescribing zinc to at least 50% of cases [data not shown]. When asked what circumstance would lead a provider to NOT recommend zinc, the most common responses were not fully understanding zinc treatment (57.9%) and not having zinc in stock (30.8%). Providers were asked to recall the duration of zinc treatment; 15.6% reported duration of at least 10 days, in adherence to the current WHO guidelines [2]. Eighty–six percent of private providers reported routinely recommending ORS as part of their practice; yet 42.7% of those could not correctly describe how to prepare ORS. Zinc and ORS were in–stock (ie, providers able to show the stocked products) for 38% and 69% of providers at the time of the interview, respectively.

A direct observation of the provider treating a child 2–59 months of age with diarrhea was conducted for 97 (42%) private providers (**Table 3**). Of the children treated during the observation session, 71% were male children and the median age was 24 months. Among the 97 providers who participated in the observations, 98% asked at least one question with regard to the history of the diarrheal episode. Zinc was sold in 16.5% of cases and caregivers were recommended to go elsewhere for zinc in an additional 13.4% of cases. Among the 28 providers who recommended zinc, 50% gave no reason as to why zinc would benefit the child and only three administered the first dose during the treatment session. Providers prescribing zinc gave variable instructions on the duration of zinc treatment; 50% recommended zinc for 10–14 days; 18% for 7 days, and 32% either recommended zinc for <7 days or did not mention duration of treatment. ORS was sold as a treatment in 56.7% of cases with an additional 20.6% instructed to purchase ORS elsewhere. Of the providers who recommended ORS, 50% gave correct preparation instructions. Providers commonly prescribed treatments other than zinc and ORS, including antibiotics (61.9%) and antidiarrheals (17.5%). Twenty–six providers (26.8%) were in compliance with current WHO/IAP guidelines and either sold or prescribed both zinc and ORS. Only 2 providers sold or prescribed zinc without ORS.

**Table 3:** Treatment behaviors of private providers observed during the treatment of a child 2–59 months of age with diarrhea (n = 97)

Provider behavior	No. (%)	
**Proportion who asked at least 1 question about the diarrhea episode:**	95 (97.9)	
Frequency of diarrhea	53 (55.8)	
Character of stool	62 (65.3)	
Duration of diarrhea	76 (80.0)	
Blood in stool	10 (10.5)	
Vomiting	10 (10.5)	
**Proportion of children given zinc**	16 (16.5)	
**Proportion of caregivers recommended to obtain zinc elsewhere**	13 (13.4)	
Place from where caregiver told to get zinc:		
Chemist	12 (92.3)	
Did not specify a particular place to go	1 (7.7)	
**Among providers who advised zinc (gave or referred, n = 28)**	
At least 1 benefit of zinc told to caregiver*	14 (50)
Reduces duration of diarrhea	1 (3.6)	
Reduces frequency of stool	6 (21.4)	
Reduces stool volume	2 (7.1)	
Good for diarrhea	8 (28.6)	
Zinc acts a tonic after diarrhea	3 (10.7)	
Proportion who demonstrated administering 1st zinc dose	3 (10.7)	
Advised to give zinc for:		
2 – 5 days	3 (10.7)	
7 days	5 (17.9)	
10 days	2 (7.1)	
14 days	12 (42.9)	
Did not advise duration	6 (21.4)	
**Proportion who sold ORS during the observation**	55 (56.7)	
Number of packets sold (n = 55):		
1	49 (89.1)	
2	6 (10.9)	
**Proportion who advised caregivers to purchase ORS**	20 (20.6)	
Benefits of ORS as told by providers to caregivers among those advised ORS (n = 71)*		
Good for rehydration/prevention of dehydration	36 (50.7)	
Good for diarrhea	12 (16.9)	
Benefits not told	26 (26.8)	
Correct method of preparation advised to caregivers	47 (66.2)	
Providers who sold or prescribed zinc and ORS	26 (26.8)	
Number of children given any feeding advice	40 (41.2)	
Proportion advised medications other than zinc and ORS	87 (89.7)	

ORS – oral rehydration salts

*Multiple responses.

## DISCUSSION

We conducted a provider assessment of 232 providers from both the formal and informal private sectors in UP, India. Private providers are the mostly widely sought sources of careseeking for pediatric diarrhea in UP; yet there is a dearth of information on private, especially informal, providers and thus it is critical to understand their knowledge and willingness to provide appropriate diarrhea treatment, including zinc and ORS. We carried out this study using short vignettes and direct observations one–year after roll–out of program implementation, by which time a pharmaceutical or NGO representative promoting and selling zinc and ORS should have visited all providers [12].

It is not surprising that we found providers were more likely to report their intention to prescribe ORS and zinc in the short vignette portion of the interview than in the observation portion of the study. This same discrepancy has been observed in Bihar, India in an evaluation of a similar program promoting zinc and ORS. Mohanan et al. found poor overall knowledge of diarrhea treatment among the 340 included providers, and none were found to treat diarrhea correctly when tested by an unannounced standard patient [8].

Despite IAP Guidelines that do not recommend antibiotics for the treatment of acute diarrhea, antibiotics remain the first line treatment for most private providers [5]. We observed consistently higher rates of antibiotic prescribing compared to zinc. In previous effectiveness studies, zinc has been shown to displace the unnecessary overuse of antibiotics [6,12], but this was not observed in our evaluations. The pharmaceutical training provided, as part of the DAZT program did not address the unnecessary use of antibiotics nor suggest that zinc could and should replace antibiotics for simple acute diarrhea. Pharmaceutical representatives may have been promoting other treatments for diarrhea in addition to zinc and ORS, such as antibiotics and antidiarrheals. Providers who prescribed zinc typically did so in addition to antibiotics and ORS. Only two providers recommended zinc in the absence of ORS, lessening concerns that NGO and pharmaceutical representatives might place more attention on zinc than life–saving ORS during drug detailing visits. Program planners employed the drug detailing approach to build rapport with providers before challenging their current practices. The visits were designed to increase confidence among informal providers, the majority of whom do not receive routine or formal training. Our results illustrate that the tendency to overprescribe antibiotics will not be changed quickly, even with the introduction of zinc. More research is warranted to determine how to best influence lasting change in prescribing practices; tactics might include group training sessions, incentives, and/or community promotion.

There were several limitations to our study, including the failure to conduct observations of all providers. It is possible that the prescribing knowledge and practices of observed providers are not representative of providers for whom an observation was not possible due to logistical constraints. We conducted the provider assessment during the dry season to facilitate the logistics of data collection, but as a consequence of this timing, diarrhea prevalence was low in some villages, resulting in less diarrhea careseeking. Our data collectors waited at each provider’s practice for one day but proceeded with the interview alone if no child eligible for observation sought care during that time frame. We also recognize that there is no gold standard method for observing providers in practice. Direct observation may bias providers to give higher quality care—a phenomenon known as the Hawthorne effect. As such, we expect the observation results to represent the best–case scenario, such that the care given to a child during an observed treatment interaction is the highest quality care the provider is able to give [10]. Lastly, our assessment is also limited by its cross–sectional nature and the failure to draw upon repeated measurements across the duration of implementation. The evaluation was originally designed to assess the knowledge and practices of providers early in the intervention and then again at the end of the program, but the plan was changed mid–course by the broader project steering committee. We maintain, however, that the results of this one–time assessment are critical because they shed light on the challenges of changing the behavior of rural providers even early in the program implementation period when training is fresh and stocks are largely in place.

The DAZT private sector model mimics the reality of how drug information is currently delivered to private providers through drug detailing visits. The expansion of drug detailing to include zinc and ORS thus represents a sustainable method, especially for reaching the informal sector with critical information and products for adequately treating childhood diarrhea. The DAZT model has also highlighted the challenges in identifying and working with informal private providers who often operate underground to avoid government penalties for practicing medicine without recognized credentials. However, despite these challenges, informal private providers are often the first choice of caregivers and thus a worthwhile target of diarrhea management programming. If substantive improvements in diarrhea treatment are to be made in rural India, improvements in the treatment practices of the informal private sector will need to be addressed by increasing access to current guidelines and providing formal and informal training opportunities. Policymakers often resist developing programs targeting the informal sector, but denying the role of informal private providers in treating the rural poor is detrimental to the overall goal of improved diarrhea treatment for young children. Programs like DAZT aim to demonstrate that informal providers, including those with little–to–no education, can be taught to adequately treat childhood diarrheal episodes with ORS and zinc and to recognize the signs and symptoms requiring referral. The low–level training provided to public sector community health workers in many countries could be expanded to cover the private sector in areas where the latter is already providing the majority of care and treatment, such as rural UP. Diarrhea remains an important cause of morbidity and mortality among young children, especially the poorest and least–served, in low– and middle–income countries. Policymakers and program planners may not be able to influence the source through which children receive care, but collaborative and targeted efforts can improve the quality of care children receive through the most frequently utilized sources, in turn improving outcomes for all young children with diarrhea.
